# Single Versus Dual Antiplatelet Therapy After Transcatheter Aortic Valve Replacement: A Meta-Analysis of Randomized Clinical Trials

**DOI:** 10.1016/j.carrev.2021.01.016

**Published:** 2022-01

**Authors:** Yousif Ahmad, James P. Howard, Mahesh V. Madhavan, Martin B. Leon, Raj R. Makkar

**Affiliations:** a Smidt Heart Institute, Cedars-Sinai Medical Center, Los Angeles, CA, USA; b National Heart and Lung Institute, Imperial College London, London, United Kingdom; c Columbia University Medical Center, New York, NY, USA; d Cardiovascular Research Foundation, New York, NY, USA

**Keywords:** TAVR, transcatheter aortic valve replacement, RCT, randomized clinical trial, SAPT, single antiplatelet therapy, DAPT, dual antiplatelet therapy, Aortic stenosis, Transcatheter aortic valve replacement, Antiplatelet therapy, Aspirin, Clopidogrel, Meta-analysis

## Abstract

**Background:**

Guidelines recommend dual antiplatelet therapy (DAPT) after transcatheter aortic valve replacement (TAVR) but guidelines predate the publication of the largest randomized trial. There have been few trials in the field to date, and with a small number of total patients; pooling their results may therefore be helpful.

**Methods:**

We systematically identified all randomized trials comparing SAPT to DAPT after TAVR. The primary endpoint was the risk of major bleeding. Secondary endpoints included all bleeding, life-threatening bleeding, stroke, myocardial infarction, death and cardiac death.

**Results:**

Four trials, randomizing 1086 participants, were eligible (541 randomized to SAPT and 545 randomized to DAPT). The weighted mean follow-up was 9.1 months. The risk of major bleeding was significantly increased after DAPT (relative risk (RR) 2.36, 95% confidence interval (CI) 1.27 to 4.40, *P* = 0.007). There was a similar increased risk for all bleeding (RR 1.65, 95% CI 1.24 to 2.19, *P* < 0.001), although not for life-threatening bleeding (RR 1.44, 95% CI 0.74 to 2.77, *P* = 0.282). There were no significant differences in the risk of stroke, myocardial infarction (MI), death or cardiac death. There was no heterogeneity observed for any endpoint (I^2^ = 0.0%).

**Conclusions:**

DAPT after TAVR is associated with an increased risk of major bleeding and all bleeding. There is no evidence of a significant difference between DAPT or SAPT for the risks of stroke, MI, death or cardiac death. However, the total number of patients randomized is small and the duration of follow-up is short. Larger scale randomized trials with longer follow-up are required to assess for any potential differences in ischemic endpoints or mortality.

## Highlights

• Guidelines recommend DAPT after TAVR but predate the publication of the largest randomized trial.
• We systematically identified all RCTs comparing SAPT to DAPT after TAVR.
• DAPT after TAVR is associated with increased risk of major all bleeding.
• Larger scale RCTs with longer follow-up are required to assess for any potential differences in ischemic endpoints or mortality.

## 1 Introduction

Transcatheter aortic valve replacement (TAVR) is a safe and effective alternative to surgical aortic valve replacement (SAVR) for patients with severe aortic stenosis, with clinical trial evidence across the spectrum of surgical risk [[1], [2], [3], [4], [5], [6]]. International guidelines recommend dual antiplatelet therapy (DAPT) following TAVR, but there is a paucity of trial data in this field. Furthermore, European and American guidelines differ in the duration of DAPT they recommend [7,8] and different valve manufacturers also recommend different durations. This has led to variability in practice in terms of the antiplatelet regimens used in clinical practice post-TAVR [9].

The recommendations for DAPT after TAVR are in part extrapolations of data from coronary stents, where prolonged DAPT has been shown to reduce ischemic complications. However, TAVR valves are larger in size and bioprosthetic in nature, and the patients receiving TAVR may generally be at increased bleeding risk due to older age and comorbidities such as renal dysfunction or hypertension. Therefore, it is apposite to determine the necessity of DAPT in TAVR patients and new clinical trial data has recently emerged [10]. There have been few trials in the field to date, and with a small number of total patients; pooling their results may therefore be helpful. We sought to perform a systematic review and meta-analysis of randomized clinical trials (RCTs) comparing single antiplatelet therapy (SAPT) to DAPT after TAVR.

## 2 Methods

The present analysis was performed according to published PRISMA guidance [11]. We prospectively registered the analysis at the PROSPERO international prospective register of systematic reviews (CRD42020208125). Ethical approval was not applicable in this case.

### 2.1 Search strategy

We performed a systematic search of the MEDLINE, Cochrane Central Register of Controlled Trials, and Embase databases from December 2010 through September 2020 for all trials comparing SAPT and DAPT after TAVR. Our search strings included (“severe aortic stenosis” OR “severe symptomatic aortic stenosis”) AND (“transcatheter aortic valve implantation” OR “transcatheter aortic valve replacement”) AND (“antiplatelet therapy”). We hand-searched the bibliographies of selected studies and meta-analyses to identify further eligible studies. Abstracts were reviewed for suitability and articles accordingly retrieved. Two independent authors performed the search and literature screening (YA and JH), with disputes resolved by consensus.

### 2.2 Inclusion criteria

Only RCTs were included, and they were eligible if they reported clinical outcomes following random allocation to SAPT or DAPT after TAVR. We did not consider observational studies. Trials comparing antiplatelet therapy to anticoagulation were not included.

### 2.3 Endpoints

The primary endpoint was the risk of major bleeding. Other endpoints included risk of all bleeding, life-threatening bleeding, major or life-threatening bleeding, minor bleeding, death, cardiac death, stroke, hemorrhagic stroke, and myocardial infarction (MI).

### 2.4 Data extraction

Two authors (YA and JH) independently abstracted the data from included trials, with disputes resolved by consensus. Tests for publication bias would only be performed in the event of 10 or more trials being suitable for inclusion [12]. Included studies were assessed using the Cochrane Risk of Bias tool [13].

### 2.5 Data analysis

Intention-to-treat analyses were used. We extracted event counts to calculate relative risks (RR). The last available follow-up time was used. Random-effects meta-analyses were performed using the restricted maximum likelihood estimator, with fixed effect as a sensitivity analysis. The I^2^ statistic was used to assess heterogeneity [14]. Low heterogeneity was defined as 0–25%; moderate heterogeneity was defined as 25–50%; and significant heterogeneity was defined as >50%. Mean values are expressed as mean ± SD unless otherwise stated. Statistical significance was set at *p* < 0.05. The statistical programming environment R [15] with the metafor package [16] was used for all statistical analyses.

## 3 Results

**Fig. 1:**
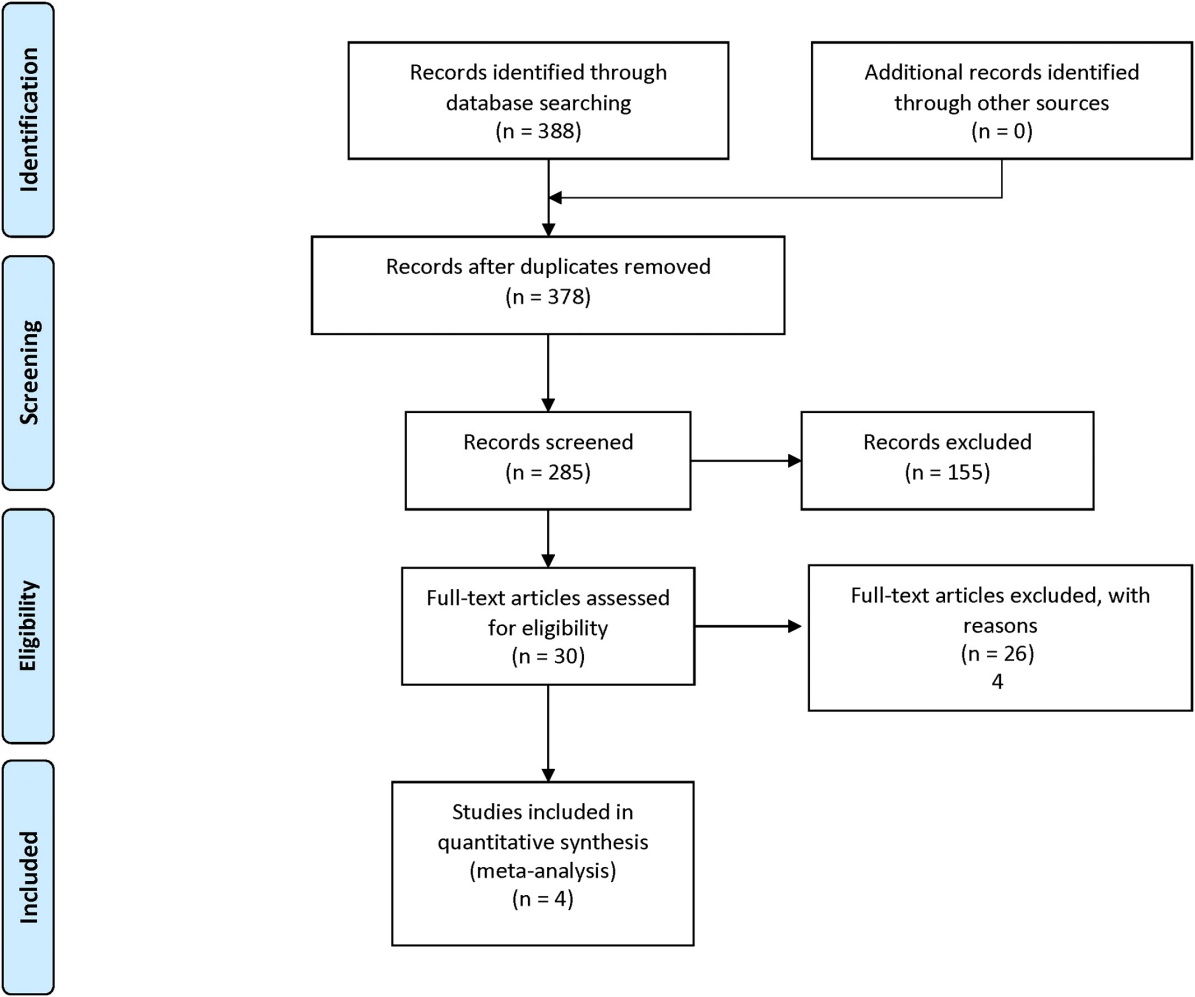
Search strategy and source of included studies.

Four trials [10,[17], [18], [19]] randomizing 1086 patients were eligible for analysis. 541 patients were randomized to SAPT and 545 patients were randomized to DAPT. Longest follow-up duration was 3 months in one trial [19], 6 months in two trials [17,18], and 12 months in one trial [10]. The weighted mean follow-up was 9.1 months. Baseline characteristics are shown in Table 1. The risk of bias assessment is shown in Table 2. The search strategy and results are shown in Fig. 1.

**Table 1:** Characteristics of included studies.

Author	Study acronym	Year	Region	N	Mean age^a^	Follow up^b^	Entry criteria	Antiplatelet regimens	TAVI type	Primary outcome
Ussia et al.		2015	Italy	79	81 (±4)	6	Consecutive patients meeting the clinical and anatomic criteria for TAVR Exclusion criteria: Previous PCI or acute coronary syndrome needing DAPT; need for oral anticoagulation; allergy or intolerance to study drugs	SAPT: aspirin alone DAPT: aspirin plus clopidogrel for 3 months	CoreValve	Composite of major adverse cardiac and cerebrovascular events (death from any cause, myocardial infarction, major stroke, urgent or emergency conversion to surgery, life-threatening bleeding)
Stabile et al.	SAT-TAVI	2014	Italy	120	81.1 (±4.8) in SAPT group 80.2 (±5.7) in DAPT group	6	Severe, symptomatic AS suitable for TAVR Key exclusion criteria: Untreated coronary artery disease requiring revascularization Acute myocardial infarction within 1 month Upper gastrointestinal bleed within 3 months CVA or TIA within 6 months Indication for oral anticoagulation therapy Aspirin/thienopridine allergy or intolerance	SAPT: aspirin alone DAPT: aspirin and clopidogrel for 6 months	Sapien XT	Not specified
Rodés-Cabau et al.	ARTE	2017	Canada, Europe, South America	222	79 (± 9)	3	Patients with clinical indication for TAVR Key exclusion criteria: Need for chronic anticoagulation Major bleeding within 3 months Prior intracranial bleed Drug-eluting stent implantation within 12 months Allergy to clopidogrel or aspirin	SAPT: aspirin alone DAPT: aspirin plus clopidogrel for 3 months	Sapien XT or Sapien 3	Composite of death, MI, ischemic stroke or TIA, or major or life-threatening bleeding at 3 months
Brouwer et al.	POPular TAVI (cohort A)	2020	Europe	665	80.4 ± 6.2 in SAPT group 79.5 ± 6.4 in DAPT group	12	Patients scheduled for TAVR without an indication for long-term oral anticoagulation Key exclusion criteria: Implantation of DES within 3 months or BMS within 1 month	SAPT: aspirin alone DAPT: aspirin plus clopidogrel for 3 months	According to local protocol	All bleeding (including minor, major, and life-threatening/disabling bleeding) at 12 months Non-procedure related bleeding at 12 months

(AS – Aortic Stenosis, TAVR – transcatheter aortic valve replacement, CVA – cerebrovascular accident, TIA – transient ischemic attack)

a Mean age ± SD given for overall population if provided; otherwise given for each group.

b Follow up in months.

**Table 2:** Risk of bias assessment.

Trial	Random sequence generation	Allocation concealment	Blinding of participants & personnel	Blinding of outcome assessment	Incomplete outcome data	Selective reporting	Overall risk of bias
Ussia et al.	Unclear Method not stated	Unclear Method not stated	High risk Un-blinded	Low risk Stated that assessment of study end points was blinded (although no further information provided)	Unclear Not stated	High risk Protocol not registered on clinicaltrials.gov	Intermediate Small randomized open-label trial with blinded assessment of study end points but no detailed description of randomization or blinding methodology.
Stabile et al.	Unclear Method not stated	Unclear Method not stated	Low risk Stated as double blind (although no further information provided)	Low risk Any clinical event adjudicated by a committee blinded to study groups	Unclear Not stated	Low risk Protocol not registered on clinicaltrials.gov	Intermdiate Small randomized trial with blinded assessment of study end points but no detailed description of randomization or blinding methodology.
Rodés-Cabau et al.	Low risk Random block sizes	Low risk Random block sizes used to conceal treatment allocation from patients	High risk Unblinded	High risk Independent clinical endpoints committee for adjudication but not blinded	Low risk No patients lost to follow-up	Intermediate risk All endpoints on clinicaltrials.gov reported. Trial prematurely stopped after 74% of planned study population because of slow enrollment	Intermediate A well conducted open-label trial with unblinded adjudication of clinical events. Trial prematurely stopped after 74% of planned study population because of slow enrollment
Brouwer et al.	Low risk Electronic web-based response system with stratification according to center	Low risk Stratification according to center	High risk Unblinded	Low risk Reported outcomes and their components were adjudicated by an independent clinical-events committee, whose members were unaware of the trial-group assignment	Low risk No missing data for any of the primary or secondary outcomes	Low risk All endpoints on clinicaltrials.gov reported	Intermediate Well-conducted randomized, open-label, investigator-initiated trial. Change from hazard ratios to risk ratios added to the statistical analysis plan as an amendment before data were unlocked (change instituted as hazards determined to be non-proportional)

In all trials, the SAPT group consisted of aspirin monotherapy. In all trials the DAPT group received aspirin plus clopidogrel in combination. In one trial, the duration of DAPT was 6 months [18], whereas in the other three the duration of DAPT was for 3 months [10,17,19]. In general, the antiplatelet agents were administered prior to the TAVR procedure. In one trial, aspirin was given at least 24 h before the procedure with clopidogrel given within 24 h before the TAVR in transfemoral cases and within 24 h after the procedure in non-transfemoral cases [19]. In another trial, aspirin was given within 1 day of the TAVR procedure, and clopidogrel was given one day before or on the day of the TAVR procedure [10]. In another trial the clopidogrel was started the day before the TAVR procedure [17], while in the final trial the information on timing was not specified [18].

### 3.1 Bleeding outcomes

**Fig. 2:**
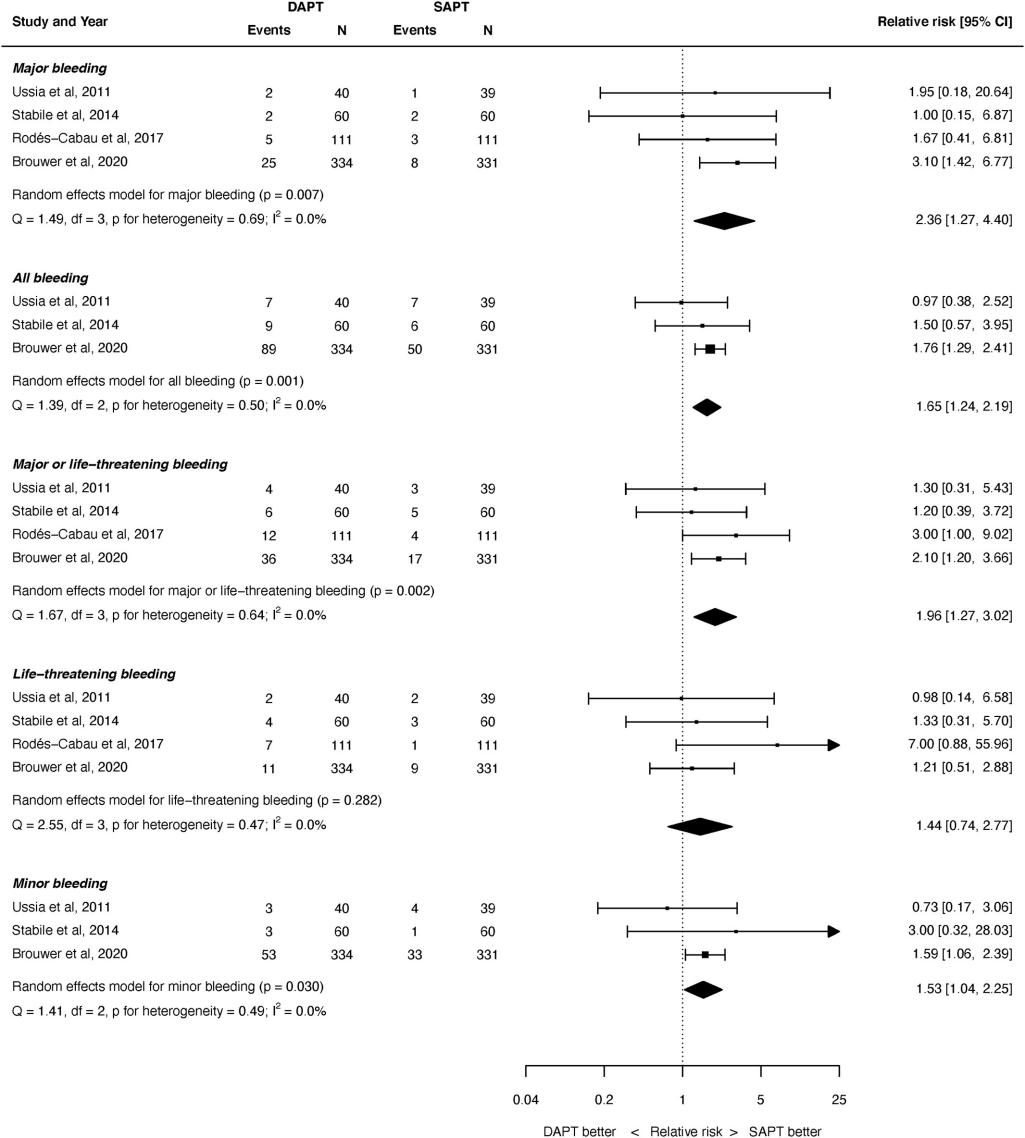
Summary of bleeding outcomes. REML = restricted maximum likelihood. Q = Cochran's Q level of heterogeneity; df = degrees of freedom.

A summary of the outcomes for the various bleeding outcomes is shown in Fig. 2. The risk of major bleeding was significantly greater with DAPT than SAPT: RR 2.36, 95% confidence interval (CI) 1.27 to 4.40, *P* = 0.007. Similarly, the risk of all bleeding was significantly greater with DAPT (RR 1.65, 95% CI 1.24 to 2.19, *P* < 0.001), as was the risk of major or life-threatening bleeding (RR 1.96, 95% 1.27 to 3.02, *P* = 0.002) and minor bleeding (RR 1.53, 95% CI 1.04 to 2.25, *P* = 0.030).. The risk of life-threatening bleeding was not significantly different after SAPT or DAPT (RR 1.44, 95% CI 0.74 to 2.77, *P* = 0.282). There was no heterogeneity for any of the bleeding outcomes (I^2^ = 0.0% for all endpoints).

### 3.2 Mortality outcomes

**Fig. 3:**
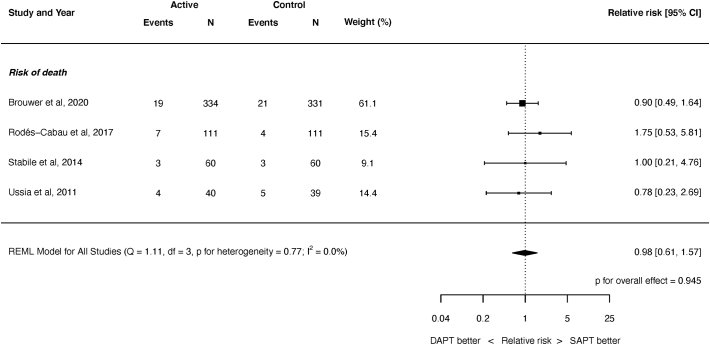
Risk of all-cause mortality. REML = restricted maximum likelihood. Q = Cochran's Q level of heterogeneity; df = degrees of freedom.

**Fig. 4:**
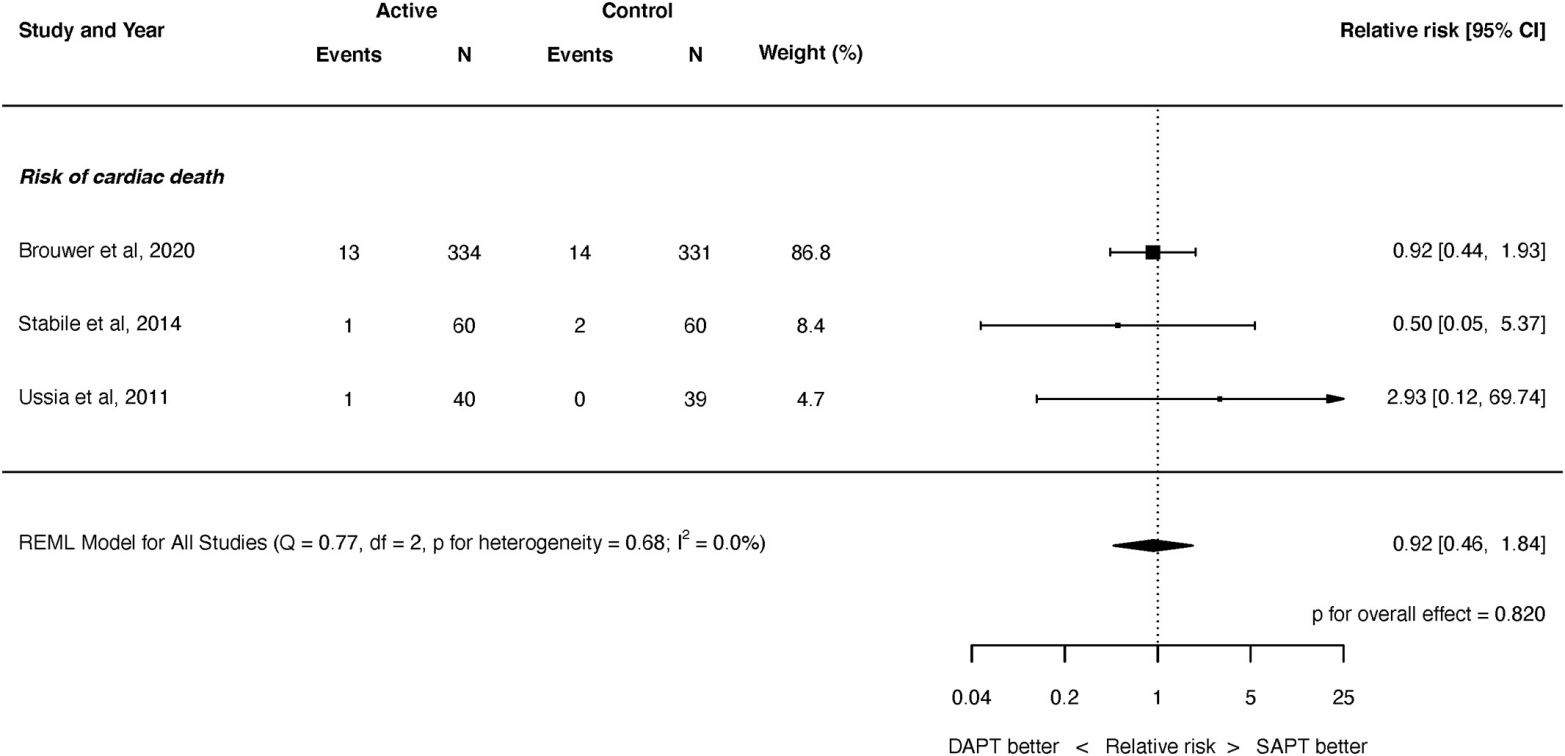
Risk of cardiac death. REML = restricted maximum likelihood. Q = Cochran's Q level of heterogeneity; df = degrees of freedom.

There was no significant difference between SAPT and DAPT for the risk of all-cause death (Fig. 3, RR 0.98, 95% 0.61 to 1.57, *P* = 0.945), or cardiac death (Fig. 4, RR 0.92, 95% CI 0.46 to 1.84, *P* = 0.820). There was no heterogeneity for either outcome (I^2^ = 0.0%).

### 3.3 Other outcomes

**Fig. 5:**
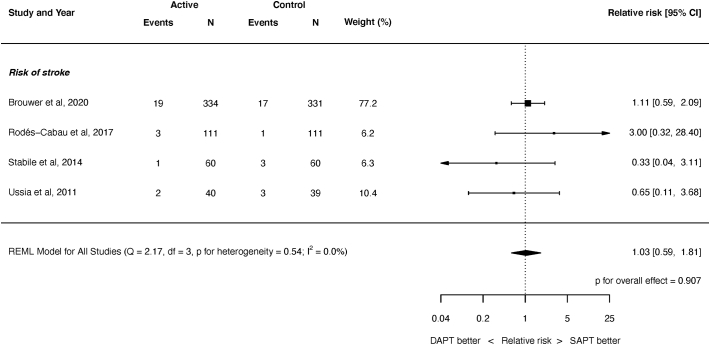
Risk of stroke. REML = restricted maximum likelihood. Q = Cochran's Q level of heterogeneity; df = degrees of freedom.

There was no significant difference between SAPT and DAPT in the risk of all stroke (Fig. 5, RR 1.04, 95% 0.59 to 1.81, *P* = 0.907), disabling stroke (RR 0.80, 95% CI 0.31 to 2.01, *P* = 0.628), hemorrhagic stroke (RR 2.99, 95% CI 0.31 to 28.48, *P* = 0.342), or myocardial infarction (RR 1.99, 95% CI 0.71 to 5.57, *P* = 0.189). There was no heterogeneity for any of the outcomes (I^2^ = 0.0% for all endpoints).

### 3.4 Sensitivity analyses

All results were consistent when analyzed by fixed effect (see Supplementary Appendix).

## 4 Discussion

In this study we have shown that the overall risk of bleeding is increased with the use of DAPT compared to SAPT after TAVR. This is manifest as a statistically significant increase in the risk of major bleeding, all bleeding, minor bleeding, and major or life-threatening bleeding. The difference in the risk of life-threatening bleeding alone was not significantly different between DAPT and SAPT, which is the only bleeding endpoint for which there was not a significant increase in risk with DAPT. There were no significant differences in the risk of death or cardiac death between the two groups, or in the risk of any ischemic/thromboembolic endpoints such as stroke or myocardial infarction. Despite the small number of trials and modest overall sample size, there was no heterogeneity observed for any endpoint in this analysis. Our analysis focuses on individual clinical endpoints rather than composite outcomes, in contrary to some prior published meta-analytic work in the field. Using composite measures in such an analysis can be problematic as each individual trial may use different composite measures as the primary outcome; therefore, meta-analysis of these outcomes is synthesizing disparate data. Taking another approach would be to count up events from individual clinical endpoints and combining them to derive a composite. If this is done, then there is a risk of counting events twice when a trial is actually providing time-to-event data.

This analysis represents the most up to date systematic review and meta-analysis of randomized trials comparing antiplatelet regimens after TAVR. It includes the recently published trial by Brouwer et al. [10] which is the largest trial in the field to date with the longest follow-up. The optimal antithrombotic regimen after TAVR remains controversial, with limited data to guide therapeutic decision making and wide variety in clinical practice protocols [9]. The rationale behind giving DAPT to patients post-TAVR is for the prevention of ischemic and thromboembolic events, in an extrapolation of data from trials of coronary stenting where DAPT has been shown to reduce the incidence of ischemic events [20,21]. However, the principles and data from coronary stenting are not necessarily applicable to TAVR with key differences both in design (much larger stent frame and bioprosthetic material in TAVR compared to metallic stents which are much smaller in coronary intervention) and patient population (patients in TAVR trials tend to be much older and with inherently greater bleeding risk than those enrolled in trials of coronary intervention).

The randomized trials comparing DAPT to SAPT after TAVR are relatively few in number and small in sample size. The largest [10] randomized a total of 665 patients, and it is also important to note that these trials generally use composite measures as their primary endpoints (or are powered for bleeding events rather than embolic events). When composite endpoints are used in clinical trials, meta-analysis can be useful to pool results and synthesize data, particularly for low-frequency but clinically important events. The results of the current analysis suggest that the increased bleeding risk with DAPT is not offset by a reduction in the risk of thromboembolic events. However, it should be noted that – even when pooling the results of all trials – the total number of events for these outcomes is low. For myocardial infarction, there were 11 events in 485 patients randomized to DAPT across three trials, and 5 events in 481 patients randomized to SAPT across three trials. Similarly, for stroke there were 25 events across 545 patients randomized to DAPT across four trials and 24 events across 541 patients randomized to SAPT across four trials. Furthermore, it may be that the pathophysiological mechanisms underpinning stroke in patients undergoing TAVR are not mitigated by DAPT. Histological studies have suggested the majority of embolic debris to the brain originate from the native aortic valve leaflets or the aortic wall [22]. Many strokes occur *peri*procedurally, and others that occur later may be related to atrial fibrillation [23], and DAPT may have a limited role for either of these potential mechanisms of stroke. Finally, another potential mechanism of stroke may be related to subclinical leaflet thrombosis, with presence of subclinical leaflet thrombosis being associated increased rates of stroke [24]. Dual antiplatelet therapy was not found to be effective in the prevention or treatment of subclinical leaflet thrombosis (whereas anticoagulation was); it therefore stands to reason that DAPT may not be effective in preventing strokes that are originating from subclinical leaflet thrombosis, but these hypotheses would all need to be tested in adequately sized randomized trials that are powered for thromboembolic events.

Current guideline recommendations for antithrombotic therapy after TAVR recommend DAPT, but these recommendations are not uniform, and are largely based on expert consensus with low strengths of recommendation. American guidelines [8,25] recommend 6 months of DAPT, and European guidelines [7] also recommend DAPT for 3–6 months, although for both of these recommendations the class of recommendation is relatively weak and the level of evidence is the lowest. On the basis of the totality of randomized trial data, pooled together in this meta-analysis, guideline recommendations may consider changing to recommend SAPT as the preferred antithrombotic regimen post-TAVR in patients with no other indication for anticoagulation.

### 4.1 Limitations

We could only report the available data, and there are only four reported trials randomizing a total of 1086 patients. The duration of DAPT was 3 months in 3 trials and 6 months in another trial. However, it is important to note that despite this there was no heterogeneity for any of the outcomes assessed in this analysis (I^2^ = 0.0% for all endpoints). We used each trial's definitions of bleeding endpoints, and considered different categories of bleeding separately to provide more granularity to the results of this analysis. Again, there was no heterogeneity observed. The follow-up duration was also not uniform across trials, with 3 months in one trial, 6 months in two trials and 12 months in another. Hazard ratios were not available for time-to-event analyses, and we therefore had to use event counts to provide relative risks as the point estimates. We were unable to perform detailed analyses of timing of events, for example to glean if the majority of events occurred early after the procedure and were related to access-site complications or were predominantly related to the clopidogrel loading dose; this data was not reported in the individual trials. There is trial data to suggest that the use of a loading dose of clopidogrel is associated with greater vascular complications [26]. This analysis does not apply to patients who have other indications for anticoagulation. Cohort B of the POPular TAVI trial [27] randomized 326 patients undergoing TAVR with an indication for anticoagulation to either no clopidogrel or clopidogrel for 3 months. The clopidogrel group had greater bleeding, mostly at the TAVR access site. Finally, our analysis only includes randomized trials which typically randomize a select minority of patients which can limit their applicability. However, randomization is the only way to compare the efficacy and safety of competing therapies without the impact of bias from both measured and unmeasured confounding factors.

## 5 Conclusions

DAPT after TAVR is associated with an increased risk of major bleeding and all bleeding. There is no evidence of a significant difference between DAPT or SAPT for the risks of stroke, MI, death or cardiac death. However, the total number of patients randomized is small and the duration of follow-up is short. Larger scale randomized trials with longer follow-up are required to assess for any potential differences in ischemic endpoints or mortality.

## Funding

The authors are grateful for infrastructural support from the National Institute for Health Research (NIHR) Biomedical Research Centre based at 10.13039/501100000762Imperial College Healthcare NHS Trust and 10.13039/501100000761Imperial College London. YA is supported by the 10.13039/501100000691Academy of Medical Sciences and 10.13039/501100013342Imperial Biomedical Research Centre. JPH is supported by the 10.13039/100010269Wellcome Trust (212183/Z/18/Z).

## CRediT authorship contribution statement

**Yousif Ahmad:** Conceptualization, Methodology, Software, Formal analysis, Data curation, Writing – original draft, Writing – review & editing, Visualization, Supervision. **James P. Howard:** Methodology, Software, Formal analysis. **Mahesh V. Madhavan:** Writing – review & editing. **Martin B. Leon:** Writing – review & editing, Supervision. **Raj R. Makkar:** Writing – review & editing, Supervision.

## Declaration of competing interest

Dr. Leon has received research support to his institution from Edwards Lifesciences, Medtronic, Boston Scientific, and Abbott; has served on Advisory Boards for Medtronic, Boston Scientific, Gore, Meril Lifescience, and Abbott; and has served as the Co-Principal Investigator of the PARTNER 3 trial (Edwards Lifesciences, no direct compensation). Dr. Makkar has received research grants from Edwards Lifesciences, Abbott, Medtronic, and Boston Scientific; has served as national Principal Investigator for Portico (Abbott) and Acurate (Boston Scientific) U.S. investigation device exemption trials; has received personal proctoring fees from Edwards Lifesciences; and has received travel support from Edwards Lifesciences, Abbott, and Boston Scientific.

## Appendix A Supplementary data

Supplementary figures Image 1
