# Supplementary material for: Single Versus Dual Antiplatelet Therapy After Transcatheter Aortic Valve Replacement: A Meta-Analysis of Randomized Clinical Trials
Source: Cardiovasc Revasc Med. 2022 Jan;34:46–53. doi: 10.1016/j.carrev.2021.01.016 (PMC8814464; doi:10.1016/j.carrev.2021.01.016)
Supplement: Supplementary file 1 — Supplementary figures [file mmc1.docx]

**Supplementary Appendix**

**Single versus dual antiplatelet therapy after transcatheter aortic valve replacement: a meta-analysis of randomized clinical trials**

**Figure 1. Risk of major bleeding when analyzed by fixed effect.**

**Figure 2. Risk of all bleeding when analyzed by fixed effect.**

**Figure 3. Risk of major or life-threatening bleeding when analyzed by fixed effect.**

**Figure 4. Risk of life-threatening bleeding when analyzed by fixed effect.**

**Figure 5. Risk of minor bleeding when analyzed by fixed effect.**

**Figure 6. Risk of all-cause mortality when analyzed by fixed effect.**

**Figure 7. Risk of cardiac death when analyzed by fixed effect.**

**Figure 8. Risk of stroke when analyzed by fixed effect.**

**Figure 9. Risk of myocardial infarction when analyzed by fixed effect.**
